# Identification of Prognostic Metabolomic Biomarkers at the Interface of Mortality and Morbidity in Pre-Existing TB Cases Infected With SARS-CoV-2

**DOI:** 10.3389/fcimb.2022.929689

**Published:** 2022-07-22

**Authors:** Ilhame Diboun, Farhan S. Cyprian, Najeha Rizwana Anwardeen, Hadi M. Yassine, Mohamed A. Elrayess, Samreen Mumtaz Rahmoon, Sarah Khaled Sayed, Sven Schuchardt, Malkan Khatib, Devendra Bansal, Elmoubashar Abu Baker Abd Farag, Mohamed M. Emara, Abdallah M. Abdallah

**Affiliations:** ^1^ Medical and Population Genomics Lab, Sidra Medicine, Doha, Qatar; ^2^ Department of Basic Medical Sciences, College of Medicine, QU Health, Qatar University, Doha, Qatar; ^3^ Biomedical Research Center (BRC), QU Health, Qatar University, Doha, Qatar; ^4^ Fraunhofer Institute for Toxicology and Experimental Medicine (ITEM), Hannover, Germany; ^5^ Department of Public Health, Ministry of Public Health, Doha, Qatar

**Keywords:** COVID-19 disease severity, tuberculosis, post-tuberculosis long-term consequences, metabolomics, biomarkers

## Abstract

Severe acute respiratory syndrome coronavirus 2 (SARS-CoV-2) infection currently remains one of the biggest global challenges that can lead to acute respiratory distress syndrome (CARDS) in severe cases. In line with this, prior pulmonary tuberculosis (TB) is a risk factor for long-term respiratory impairment. Post-TB lung dysfunction often goes unrecognized, despite its relatively high prevalence and its association with reduced quality of life. In this study, we used a metabolomics analysis to identify potential biomarkers that aid in the prognosis of COVID-19 morbidity and mortality in post-TB infected patients. This analysis involved blood samples from 155 SARS-CoV-2 infected adults, of which 23 had a previous diagnosis of TB (post-TB), while 132 did not have a prior or current TB infection. Our analysis indicated that the vast majority (~92%) of post-TB individuals showed severe SARS-CoV-2 infection, required intensive oxygen support with a significantly high mortality rate (52.2%). Amongst individuals with severe COVID-19 symptoms, we report a significant decline in the levels of amino acids, notably the branched chains amino acids (BCAAs), more so in the post-TB cohort (FDR <= 0.05) in comparison to mild and asymptomatic cases. Indeed, we identified betaine and BCAAs as potential prognostic metabolic biomarkers of severity and mortality, respectively, in COVID-19 patients who have been exposed to TB. Moreover, we identified serum alanine as an important metabolite at the interface of severity and mortality. Hence, our data associated COVID-19 mortality and morbidity with a long-term metabolically driven consequence of TB infection. In summary, our study provides evidence for a higher mortality rate among COVID-19 infection patients who have history of prior TB infection diagnosis, which mandates validation in larger population cohorts.

## Introduction

SARS-CoV-2 remains the most challenging pathogen at present; although, *Mycobacterium tuberculosis* (*M. tuberculosis*), the causative agent of tuberculosis (TB), is one of the oldest pathogen that has evolved and adapted to afflict humans for over 9000 years (CDC, 2016) remains the most serious infectious disease prior to COVID-19 (Crisan-Dabija et al., 2020). *M. tuberculosis* is an intracellular pathogen that causes an enormous worldwide burden of disease, and is still one of the major causes of morbidity and mortality. As a central aspect of its pathogenesis, *M. tuberculosis* grows in lung alveolar macrophages, and host and microbe influence each other’s metabolism. Hence, a notable feature of TB infection is its striking heterogeneity on lung function that ranges from no impairment to severe dysfunction (Plit et al., 1998; Ralph et al., 2013; Mahajan et al., 2020) as well as other types of ventilatory defects (Willcox and Ferguson, 1989; Plit et al., 1998; Byrne et al., 2015). Indeed, patients who are successfully cured of TB with antibiotics may present with cavitation, nodular infiltrates or fibrosis, or may have a mix of these pulmonary pathologies and more than one-third of them may developed permanent lung damage (Long et al., 1998; Hunter, 2011). This enormous variability may relate to host–pathogen interactions and the varied immunological events that can follow. Despite the shift in attention from TB to COVID-19, TB remains a huge threat because of its long-term consequences in COVID-19 patients. Since both infections mainly target lung tissues, patients with TB history are at high risk to develop more severe effects relating to COVID-19 (Mcquaid et al., 2020; Wingfield et al., 2020). These effects are due to the long-term consequence of TB, including compromised lung tissue (Halezeroglu et al., 1997; Eren et al., 2003), post-tuberculosis lung damage (PTLD) (Meghji et al., 2020), acceleration of lung aging (Van Zyl Smit et al., 2010) and chronic obstructive pulmonary disease (COPD) (Amaral et al., 2015; Byrne et al., 2015). The infection with *M. tuberculosis* is accompanied by a significant, well-documented metabolic changes; evident in the loss of lean tissue in these patients (Paton and Ng, 2006).

Wide scale metabolomics analysis has deepened our understanding of biological mechanisms involved in several noninfectious diseases and provided a platform for the identification of new biomarkers (Kaddurah-Daouk et al., 2008). Metabolic analysis has provided valuable diagnostic and therapeutic insight, particularly in long standing disorders such as insulin resistance, type 2 diabetes (Guasch-Ferre et al., 2016; Al-Sulaiti et al., 2018; Al-Sulaiti et al., 2019), cancer (Sibal et al., 2010; Peitzsch et al., 2022) cardiovascular disease (Barderas et al., 2011; Zhang et al., 2015; Paapstel and Kals, 2022) as well as infections like COVID-19 (Maras et al., 2021). In the context of infectious diseases, several metabolomics analyses have demonstrated that lipid and amino acid metabolism of the human host is altered in mycobacterial diseases that may leave an imprint post recovery (Al-Mubarak et al., 2011; Weiner et al., 2012; Mahapatra et al., 2014; Mayboroda et al., 2016; Silva et al., 2017). Recent studies have identified metabolic signatures associated with COVID-19 severity (Shen et al., 2020; Thomas et al., 2020) and outcome at intensive care units (Taleb et al., 2021). *M. tuberculosis* and Sars-CoV-2 infections independently alter the host metabolism, we therefore, we hypothesized that COVID-19 patients, who were previously infected with TB would exhibit metabolic differences in comparison with COVID-19 patients that were not previously infected with TB.

Here, we investigated the effect of history of TB infection on metabolic profiles and associated COVID-19 outcome relying on targeted metabolomics. We uncover a metabolic signature of pro-inflammatory macrophages in patients with TB history who tend to present severe cases of COVID-19 infection as opposed to a potentiation of anti-inflammatory processes in control group.

## Materials and Methods

### Study Design

This is a cross-sectional study involving 155 adult patients infected with SARS-CoV-2, diagnosed by the public health care provider (Hamad Medical Corporation - HMC) in Qatar, between July 2020 and August 2021. The patients included in the study were identified *via* public screening programs or hospital admission. All cases were diagnosed in labs reporting to public health care facilities using RT-PCR, employing TaqPath COVID-19 Combo Kit (Thermo Fisher Scientific, Waltham, Massachusetts) or Cobas SARS-CoV-2 Test (Roche Diagnostics, Rotkreuz, Switzerland) from upper respiratory tract specimens (throat and nasopharyngeal swabs). Consented patients provided 2 -3 ml of venous blood samples in serum tubes collected at the time of diagnosis, prior to isolation, or during hospitalization. The study cohort included patients of several nationalities resident in Qatar and were clinically identified as symptomatic, mild, moderate, severe, or critical based on COVID-19 WHO guidelines (WHO, 2021). For analysis, the patients were clustered into three groups as asymptomatic, mild (includes WHO mild and moderate, not requiring oxygen support) and severe (incorporates WHO critical and severe cases on supplemental oxygen with or without ventilatory support). The group of COVID-19 patients with mild to severe disease were hospitalized for inpatient management, out of which 31 patients died with respiratory failure listed as the primary cause of death. Standard of care for hospitalized patients consisted of supportive care and antiviral therapy, with individual regimens selected based on the severity of disease, the presence of comorbidities, contraindications, or potential drug-drug interactions. Clinical and demographic data including anthropometrics, and medical history data including COVID-19 disease severity were collected from HMC’s electronic healthcare system following consent of patients. Our recruited COVID-19 patients were dichotomized into those with prior diagnosis of TB (n=23, referred in this study as *post-TB*), and *controls* defined as those who did not experience pulmonary TB in the past (n=132). We had limited access to female patients (n=16) due to predominant male infectivity and other constraints during sample collection. The participants were randomly selected and therefore the total cohort had a low representation of post-TB cases (n=23) (**Table 1**). All protocols were approved by Institutional Review Boards (IRBs) of HMC (MRC-05-136) and Qatar University (QU-IRB 1418-EA/20).

**Table 1:** Clinical traits of participants stratified by mild, moderate and severe Covid-19 cases.

	Severity				*p*-values		
	Total N = 155	Asymptomatic N = 36	Mild N = 55	Severe N = 64	Severe vs. Mild	Severe vs. Asymptomatic	Mild vs. Asymptomatic
**Age**	55 (48-63)	52 (46-55)	51 (46-58)	63 (55-70)	<0.001	<0.001	0.8
**Body Mass Index**	28.2 (25.5-31.9)	26.1 (23.9-27.7)	28.6 (26.1-32.6)	28.3 (25.5-31.0)	0.8	0.11	0.072
**Gender**					0.089	0.4	0.045
Male	139 (100%)	35 (25%)	45 (32%)	59 (42%)			
Female	16 (100%)	1 (6.2%)	10 (62%)	5 (31%)			
**TB Status**					<0.001	0.006	0.062
Post-TB	23 (100%)	2 (8.7%)	0 (0%)	21 (91%)			
BCG vaccination (+)	49 (100%)	10 (20%)	28 (57%)	11 (22%)			
BCG vaccination (-)	18 (100%)	6 (33%)	8 (44%)	4 (22%)			
Unknown	65 (100%)	18 (28%)	19 (29%)	28 (43%)			
**Living status**					<0.001	<0.001	0.053
**Alive**	123 (79.4%)						
Post-TB	12 (100%)	2 (17%)	0 (0%)	10 (83%)			
BCG vaccination (+)	47 (100%)	10 (21%)	28 (60%)	9 (19%)			
BCG vaccination (-)	18 (100%)	6 (33%)	8 (44%)	4 (22%)			
Unknown	46 (100%)	18 (39%)	18 (39%)	10 (22%)			
**Dead**	32 (20.6%)						
Post-TB	11 (100%)	0 (0%)	0 (0%)	11 (100%)			
BCG vaccination (+)	2 (100%)	0 (0%)	0 (0%)	2 (100%)			
Unknown	19 (100%)	0 (0%)	1 (5.3%)	18 (95%)			
**Comorbidities**							
**Diabetes mellitus**					>0.9	0.040	0.047
Yes	77 (100%)	12 (16%)	30 (39%)	35 (45%)			
No	78 (100%)	24 (31%)	25 (32%)	29 (37%)			
**Cardiovascular disease**					0.030	0.013	0.5
Yes	12 (100%)	0 (0%)	2 (17%)	10 (83%)			
No	143 (100%)	36 (25%)	53 (37%)	54 (38%)			
**Asthma**					>0.9	0.2	0.15
Yes	9 (100%)	0 (0%)	4 (44%)	5 (56%)			
No	146 (100%)	36 (25%)	51 (35%)	59 (40%)			

Parametric traits are described with mean ± sd, non-parametric using median (IQR) whilst categorical variables are given in counts. F-test/Tukey’s HSD, Pairwise Mann Whitney U, Fisher’s exact test are used as appropriate.

### Metabolomics

Targeted metabolomics of serum samples from participants was conducted using Biocrates MxP^®^ Quant 500 Kit (Biocrates, Innsbruck, Austria) measured by tandem mass spectrometry at Fraunhofer Institute for Toxicology and Experimental Medicine. Six hundred and thirty metabolites were assessed as part of the MetIDQ™ MetaboINDICATOR™ module designed specifically for MxP^®^ Quant 500 kit data. A total of 232 metabolite indicators, consisting of combinations of metabolite measurements that capture meaningful biological functions, were also derived. Examples of such indicators include enzymatic activity based on substrate/product metabolite ratio and the sum of functionally/structurally similar metabolites. A full list of metabolite indicators can be found in **Supplementary File 1**. Flow Injection Analysis Tandem Mass Spectrometry (FIA-MS/MS) was used to quantify lipids and liquid chromatography–mass spectrometry (LC-MS/MS) was used to quantify small molecules using 5500 QTRAP^®^ instrument (SCIEX Darmstadt, Germany) as previously described (Mahajan et al., 2020).

### Statistical Analysis: Linear Models

Metabolomics data were log-transformed. As a part of quality assurance, two subjects deemed outliers in PCA analysis were excluded from further analysis. Multivariate analysis including non-supervised PCA and supervised OPLS-DA (Orthogonal Projection to Latent Structures Discriminant Analysis) were run using the software SIMCA^®^ (version 16). With both analyses, metabolic metabolite indicator measurements were excluded to avoid bias owing to possibly differing distributional properties in comparison to the original metabolite measurements. R version 4.0.2 was used to run a linear model per metabolite (featuring as the y-variable) versus post-TB (post-TB/controls), living status (alive/dead) as well as the interaction between them. The model also featured the following confounders: age, gender, BMI, cardiovascular disease, and diabetes. R package Emmeans was used to assess the differences in marginal means between post-TB and control individuals stratified by living status. Another linear model, featuring the same confounders, was run to assess metabolites’ association between post-TB versus controls, both groups showing severe levels of Covid-19 infection. Nominal *p*-values were penalized for multiple testing using the false discovery rate (FDR) procedure.

### Enrichment Analysis

Enrichment analysis of functional/structural classes was run on all *p*-value ordered metabolite lists from the Linear Models and regression analyses previously outlined. This was based on the one-way Wilcoxon rank sum test, followed by FDR based multiple testing correction. The classes were predefined by Biocrates and those with less than three members were disqualified. Furthermore, metabolite indicator sums and ratios were excluded from enrichment analysis.

### Comparison With Published Data Featuring Characterized Cell Phenotype

Metabolomics data measured from M1 inflammatory and M2 anti-inflammatory macrophages, featuring five replicates from each group, were obtained from a published omics study by Jha et al. Briefly (Jha et al., 2015), bone marrow cells were extracted from a single mouse and differentiated into M1/M2 polarized macrophages by exposure to either lipolysaccharides (LPS) and IFN-γ or IL4. The data were subjected to QC and all ten samples were retained. The published dataset in question and our measured data were separately scaled to have a mean of zero and a variance of 1 for direct comparison. To allow mapping of metabolites between the two datasets, our measured metabolites were mapped to HMDB entries using MetaboAnalyst (Chong and Xia, 2020). Fifty-one metabolites were common to both datasets. A SIMCA OPLS-DA classifier was trained on the published dataset to recognize the differences in these metabolites between the M1 versus M2 macrophages. The model was then used to predict our study groups: COVID-19 control and COVID-19 vulnerable post-TB. Another model was trained on our measured data and used to tell apart the polarized macrophages from the published study. Roc curve analysis combined with the area under the curve (AUC) metric were used for assessment of the predictive capacity of the models.

### Network Partial Correlations

Partial correlations were calculated using the R package GeneNet (Taylor et al., 2015) based on the Gaussian graphical models (GGMs) using metabolite data measured from the controls. The FDR significant partial correlations obtained with the GGMs were displayed as edges linking the respective pairs of metabolites, together giving rise to an overview of the metabolic network in the blood of control subjects. GGM partial correlations scoring an FDR corrected *p*-value of 6.56 10^-14^ were collectively displayed in a network using the software Cytoskape (version 3.7.2) (Kohl et al., 2011). The nodes in the network capturing the correlated metabolites were color-coded to reflect the changes in the post-TB groups with respect to control as main effects averaged across all COVID-19 severity groups. The nodes were given different shapes to reflect the type of the given metabolite.

## Results

### COVID-19 Mortality Is Higher in Patients who Have Recovered From TB Infection

In this study, targeted metabolome from peripheral blood of 155 adult COVID-19 patients was measured, notably 23 individuals had a confirmed diagnosis of pulmonary TB infection in the past (post-TB) while 132 controls did not have a history of TB infection. The subjects were classified according to WHO guidelines of COVID-19 severity and history of TB or BCG vaccination (**Table 1**). Similar to reported literature, age and cardiovascular diseases were risk factors for severe COVID-19 in our cohort. As expected, the COVID-19 patients showed an increase in the death incidence amongst the severe group as compared to mild and asymptomatic COVID-19 patients (*p*-value <0.001) (**Table 1**). Importantly, we noted a significantly higher incidence of previous infection with TB amongst the severe cases in comparison to mild (*p*-value <0.001) (**Table 1**). Interestingly, the severe cases, who had been diagnosed with TB in the past, demonstrated the highest mortality rate among all SARS-CoV-2 infected patients (**Table 1**).

### Multivariate Analysis Showed Marked Variation in Metabolic Profile Between Controls and Post-TB Groups

**Figure 1:**
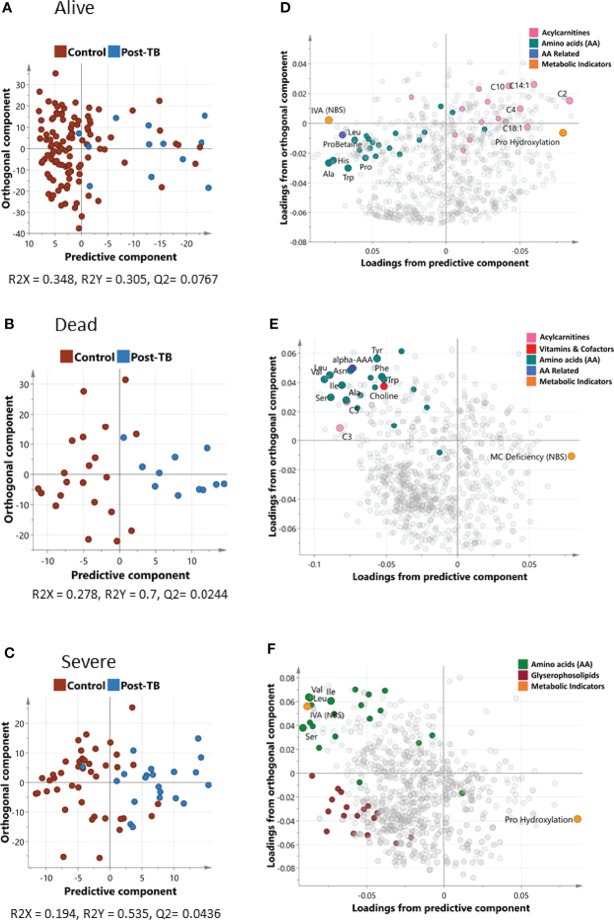
Orthogonal Projections to Latent Structures Discriminant Analysis (OPLS-DA) of controls and post-TB. The analysis was run on alive and dead patient cohorts separately represented by **(A, B)** that show the score plots for the two groups respectively. Both OPLS-DA models identified one predictive and one orthogonal component. **(D, E)** show the loading plots for alive and dead groups respectively. OPLS-DA of controls/post-TB was also performed on covid-19 patients with severe symptoms **(C, F** depicts the score and loadings plot respectively**)**. Metabolites with the most discrepant loadings are labelled and their respective categories highlighted in color. It is important to note that the predictive components in OPLS-DA are associated with controls/post-TB as opposed to the orthogonal component, which captures hidden confounder effects. IVA (NBS) is the ratio C5/C2, Pro Hydroxylation is (c4-OH-Pro + t4-OH-Pro)/Proline, MC Deficiency (NBS) is C16/C3, more details can be found in **Supplementary File 1**.

Using OPLS-DA supervised multivariate analysis, we captured the joint metabolic signature of post-TB versus controls amongst COVID-19 patients who survived or failed to survive (**Figures 1A, B** respectively). For both groups, one predictive component, and one orthogonal component were observed, although a higher degree of separation of post-TB versus controls was observed in deceased patients (the explained post-TB/controls variance R^2^ was 0.7/0.305 for dead/alive groups respectively). Since most post-TB subjects also suffered from severely COVID-19, we performed an additional OPLS-DA analysis looking at differential metabolic signature of post-TB versus controls amongst severe subjects only. A reasonable separation was observed (R^2^_=_ 0.535) indicating metabolic alterations in the post-TB group in equally severely affected patients (**Figure 1C**). Overall, the metabolic map underlying the post-TB and controls groups' separation show decreased levels of amino acids amongst the post-TB and an increased hydroxylation of proline amongst the post-TB, in all three comparisons (**Figures 1D–F**). Conversely, the metabolism of acyl-carnitines and glycerophospholipids seem to bear a differential value for survival and COVID-19 severity (**Figures 1D, F** respectively).

### Univariate Analysis Indicates Differential Post-TB/Controls Signature in Alive Versus Deceased Patients

**Figure 2:**
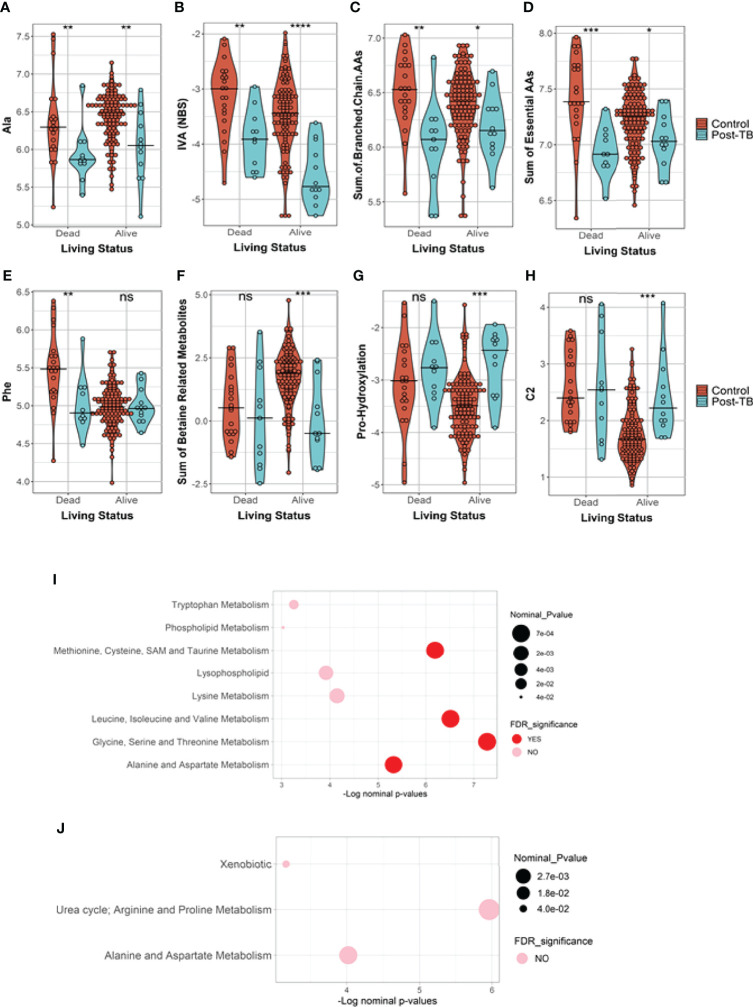
Metabolites and metabolic indicators have differential levels in post-TB Covid-19 infected subjects in comparison to controls. The results are stratified based on survival status where, **(A, B)** show metabolites similarly reduced in post-TB in alive and dead subjects. **(C–E)** are metabolites and metabolic indicators significantly changed amongst the deceased subjects only whilst **(F–H)** show instances of significant metabolite changes unique to the survivors group (FDR<=0.05, ***). **(I, J)** Indicate the functional enrichment analysis performed on the ranked list of metabolites from dead/alive subjects respectively. Ns indicates nominal *p*-values that did not pass the FDR cut-off of 0.05. The line across the violins indicates the median in each group. Definitions of the metabolite indicators can be found in **Supplementary File 1**.

Stratified association analysis per survival group revealed similar and differential changes in metabolite levels between controls versus post-TB. At the FDR <= 0.05, the amino acid alanine was significantly depleted in the post-TB group regardless of survival outcome (**Figure 2A**) similar to the metabolic indicator isovaleric acid - IVA (NBS), (**Supplementary File 1**), although at a less stringent level of significance (**Figure 2B**). Effects uniquely observed among COVID-19 non-survivors include significantly lower levels of aromatic amino acids, branched chain amino acids, essential and non-essential amino acids, and glucogenic and ketogenic amino acids in post-TB patients as compared to the controls (FDR <= 0.05) (**Figures 2C–E** and **Table 2**). On the other hand, increased proline hydroxylation, elevated acetylcarnitine C2 levels and depleted betaine and derivatives in the post-TB group were only observed amongst survivor patients (**Figures 2F–H** and **Table 3**). The enrichment analysis further substantiated these results by revealing the importance of ‘methionine, cysteine, SAM and taurine metabolism’, ‘leucine, isoleucine and valine metabolism’, ‘glycine, serine and threonine metabolism’ and ‘alanine and aspartate metabolism’ amongst COVID-19 deceased as opposed to urea, arginine and proline metabolism amongst COVID-19 survivors (**Figures 2E, J**). Interestingly, these observations are in line with the results obtained from the multivariate analysis (**Figures 1D, E**). The full list of FDR <= 0.05 metabolites from comparing controls versus post-TB in alive/dead Covid-19 subjects can be found in **Tables 2** and **3** respectively.

**Table 2:** Top FDR hits from linear model analysis of metabolite levels in controls versus post-TB among dead COVID-19 patients.

Metabolite/Metabolism indicator	Definition	Living Status	Effect^#^	SE	P value	Fdr
Val	Amino acids	Dead	0.68	0.13	1.33E-06	0.0010
Sum of Branched Chain AAs*	Ile + Leu + Val	Dead	0.69	0.14	3.22E-06	0.0012
Leu	BCAA^%^	Dead	0.73	0.15	5.12E-06	0.0013
Sum of Essential AAs*	His + Ile + Leu + Lys + Met + Phe + Thr + Trp + Val	Dead	0.54	0.12	1.82E-05	0.0035
Sum of Aromatic AAs*	Phe + Trp + Tyr	Dead	0.49	0.12	6.79E-05	0.0083
Ile	BCAA	Dead	0.65	0.16	6.80E-05	0.0083
Sum of AAs*	Sum of AAs	Dead	0.42	0.10	7.51E-05	0.0083
Sum of Solely Ketogenic AAs*	Leu + Lys	Dead	0.57	0.14	1.24E-04	0.0116
Sum of Solely Glucogenic AAs*	Sum of Solely Glucogenic AAs	Dead	0.38	0.10	2.58E-04	0.0194
C5	Acylcarnitines	Dead	0.96	0.25	2.75E-04	0.0194
Phe	Amino acids	Dead	0.49	0.13	3.61E-04	0.0227
Ratio of PC ae to Choline*	Ratio of Acyl-Alkyl-Phosphatidylcholines to Choline	Dead	-0.65	0.18	3.81E-04	0.0227
Sum of Non-Essential AAs*	Sum of Non-Essential AAs	Dead	0.35	0.10	6.61E-04	0.0335
Ala	Amino acids	Dead	0.57	0.16	6.69E-04	0.0335
Asn	Amino acids	Dead	0.46	0.13	6.92E-04	0.0335
Tyr	Amino acids	Dead	0.53	0.15	9.17E-04	0.0418
Ratio of PCs to Choline*	Ratio of Phosphatidylcholines to Choline	Dead	-0.58	0.17	1.07E-03	0.0458
Alpha-AAA	2-aminoadipate	Dead	0.84	0.25	1.18E-03	0.0481

*Indicates a metabolic indicator derived from combination of original metabolite measurements (**Supplementary File 1**)

The effect represents the offset in the intercept from the controls baseline to post-TB individuals, all subjects are deceased.

^%^ BCAA refer to branched chains amino acids. ^#^The effect represents the offset in the intercept from the controls baseline to post-TB individuals, all subjects are deceased.

**Table 3:** Top FDR hits from linear model analysis of metabolite levels in controls versus post-TB among survivor COVID-19 patients.

Metabolite/Metabolism indicator	Definition	Living Status	Effect^#^	SE	P value	FDR
IVA NBS*	C5/C2	Alive	1.096	0.25	2.42E-05	0.015
SBCAD Deficiency NBS*	C5/C0	Alive	0.78	0.18	3.74E-05	0.015
Sum of Betaine Related Metabolites*	Sum of Betaine Related Metabolites	Alive	1.95	0.47	6.63E-05	0.015
Pro Hydroxylation*	(c4-OH-Pro + t4-OH-Pro)/Pro	Alive	-0.94	0.23	8.64E-05	0.015
Pro Betaine	Amino acids Related	Alive	2.35	0.58	9.89E-05	0.015
His	Amino acids	Alive	0.38	0.09	1.43E-04	0.018
Ala	Amino acids	Alive	0.48	0.13	4.21E-04	0.046
Ratio of SM-OHs to SM-Non Ohs*	SM (OH) Cxx:x/SM Cxx:x	Alive	0.22	0.06	5.47E-04	0.046
OTC Deficiency NBS*	Orn/Cit	Alive	0.73	0.21	6.10E-04	0.046
Cit Synthesis*	Cit/Orn	Alive	-0.73	0.21	6.32E-04	0.046
C2	Acylcarnitines	Alive	-0.7	0.19	6.56E-04	0.046
2 MBG NBS*	C5/C3	Alive	0.71	0.2	8.12E-04	0.053

*Indicates a metabolic indicator derived from combination of original metabolite measurements (**Supplementary File 1**)

The effect represents the offset in the intercept from the controls baseline to post-TB individuals. **All subjects are alive.**

^%^BCAA refer to branched chains amino acids. ^#^The effect represents the offset in the intercept from the controls baseline to post-TB individuals, all subjects are deceased.

**Figure 3:**
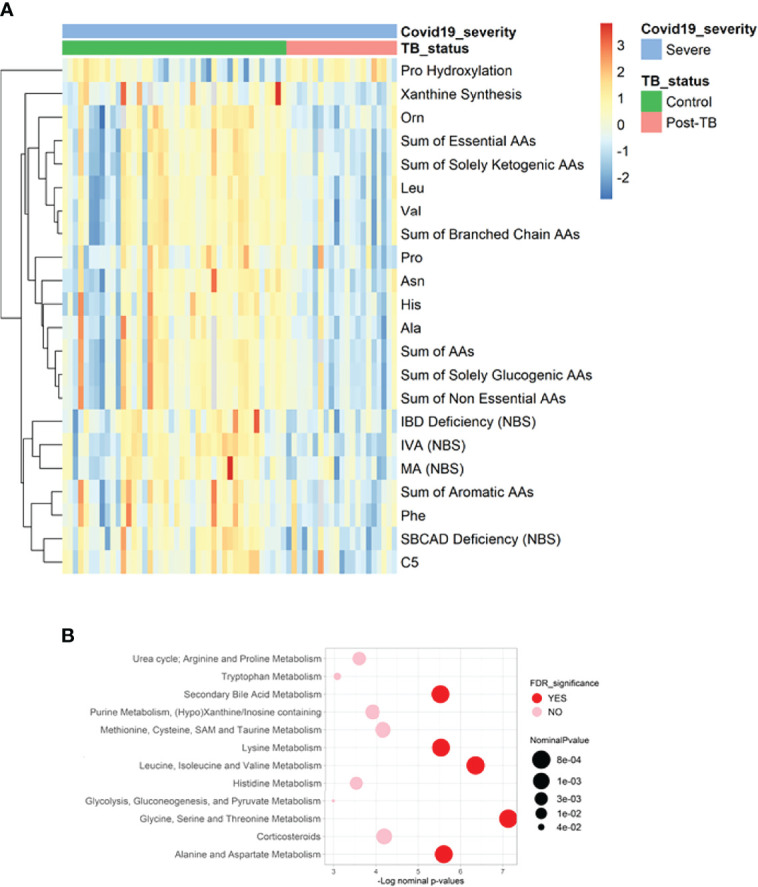
Heatmap and functional enrichment analysis from comparing post-TB and control COVID-19 patients with severe symptoms. **(A)** Heatmap representing the metabolites with significantly different levels (FDR<=0.05). **(B)** Results from functional enrichment analysis based on metabolite ranks by *p*-value using the Wilcoxon rank sum test. Definitions of the metabolite indicators can be found in **Supplementary File 1**.

Comparative analysis of metabolite levels between post-TB and controls among severe COVID-19 cohort revealed changes akin to previously noted within the stratified analyses (**Figure 3** and **Table 4**). An exception was noted with the increase in xanthine synthesis in the controls in comparison to post-TB, not observed in previous analyses.

**Table 4:** Top FDR hits from linear model analysis of metabolite levels in controls versus post-TB amongst severely affected Covid-19 cohort.

Metabolite/Metabolite Indicator	Definition	Covid_19 Severity level	Effect	Standard Error	P value	FDR
IVA NBS*	C5/C2	Severe	0.913	0.20	2.56E-05	0.012
Val	BCAA^%^	Severe	0.400	0.09	3.03E-05	0.012
Leu	BCAA	Severe	0.441	0.10	5.16E-05	0.013
Sum of Essential AAs*	Sum of Essential AAs	Severe	0.344	0.08	8.60E-05	0.012
Sum of Branched Chain AAs*	Ile + Leu + Val	Severe	0.397	0.09	8.82E-05	0.012
SBCAD Deficiency NBS*	C5/C0	Severe	0.603	0.15	1.63E-04	0.019
Ala	Amino acids	Severe	0.412	0.10	1.75E-04	0.019
Sum of AAs*	Sum of AAs	Severe	0.268	0.07	2.42E-04	0.022
Pro Hydroxylation*	(c4-OH-Pro + t4-OH-Pro)/Pro	Severe	-0.709	0.18	2.50E-04	0.022
Xanthine Synthesis*	Xanthine/Hypoxanthine	Severe	0.801	0.21	3.17E-04	0.025
Sum of Aromatic AAs*	Phe + Trp + Tyr	Severe	0.308	0.08	3.66E-04	0.025
Phe	Amino acids	Severe	0.330	0.10	4.34E-04	0.026
Sum of Solely Glucogenic AAs*	Sum of Solely Glucogenic AAs	Severe	0.249	0.07	4.48E-04	0.026
Asn	Amino acids	Severe	0.318	0.089	4.96E-04	0.026
Pro	Amino acids	Severe	0.413	0.11	5.23E-04	0.026
Sum of Solely Ketogenic AAs*	Leu + Lys	Severe	0.353	0.10	5.59E-04	0.026
C5	Acylcarnitines	Severe	0.663	0.19	5.68E-04	0.026
His	Amino acids	Severe	0.262	0.07	7.62E-04	0.032
Orn	Amino acids	Severe	0.379	0.11	7.79E-04	0.032
IBD Deficiency NBS*	C4/C2	Severe	0.609	0.18	8.46E-04	0.032
MA NBS*	C3/C2	Severe	0.663	0.19	1.01E-03	0.035
Sum of Non-Essential AAs*	Sum of Non-Essential AAs	Severe	0.226	0.07	1.16E-03	0.039

*Indicates a metabolic indicator derived from combination of original metabolite measurements (**Supplementary File**)

The effect represents the offset in the intercept from the controls baseline to post-TB individuals, all subjects showing equally severe symptoms of Covid-19 infection.

^%^BCAA refer to branched chains amino acids.

### Comparison With Published Metabolomics Dataset Featuring Pro Versus Anti-Inflammatory Macrophages

**Figure 4:**
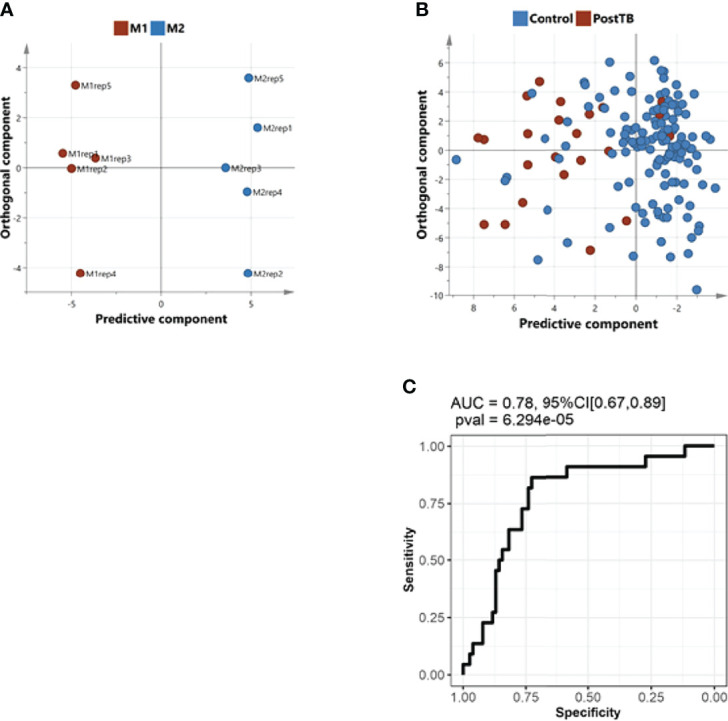
Comparison of the metabolic signature of controls/post-TB subjects with the metabolic potentiation of pro-inflammatory macrophage phenotype. **(A)**, An OPLS-DA multivariate classifier of pro-inflammatory macrophages (M1) and anti-inflammatory macrophages (M2), (data obtained from published literature, refer to methods) **(B)** The model in A was used to predict the controls/post-TB affiliation of our subjects. **(C)** ROC curve analysis indicating a concordance between the metabolic signature underlying macrophages’ switch from anti to proinflammatory phenotype and the metabolic alterations in post-TB in comparison to controls. The analysis suggests that the immune response in post-TB patients is pro-inflammatory.

Several studies have confirmed an overactive immune system in severe COVID-19 patients that correlates with patient mortality. Infections with TB reprogram the macrophages to secrete a distinct set of cytokines (Herzmann et al., 2017). Moreover, macrophages subpopulations have been identified to exert a possible anti-inflammatory or pro- inflammatory response that is identifiable by their distinct metabolomics signature. To investigate this further, we sought a direct comparison with a published metabolomics dataset featuring macrophages experimentally induced to adopt the inflammatory (M1) versus the anti-inflammatory (M2) phenotypes. Statistically, we performed this comparison by initially learning the multivariate signature of M1 versus M2 macrophage subpopulations (**Figure 4A**), and then showing that this signature effectively discriminates control and post-TB study groups (**Figure 4B**), and ROC curve analysis further confirmed the concordance between the two contrasts (**Figure 4C**, AUC=0.78, *p*-value= 6.23e-^05^). The *in-silico* analysis demonstrated a predominant trend of M1 macrophage metabolic signature in post-TB COVID-19 patients that showed the highest mortality rate among all groups (**Figure 4B**). These results indicate that the inflammatory M1 phenotype is predominantly associated with post-TB patients who died from SARS-CoV2, whereas the anti-inflammatory M2 is mainly linked to the control group, which showed higher survival rate who had higher survival.

### Network-Based Correlation Analysis Demonstrated Metabolites Associated With the High Mortality Status in the Post-TB

**Figure 5:**
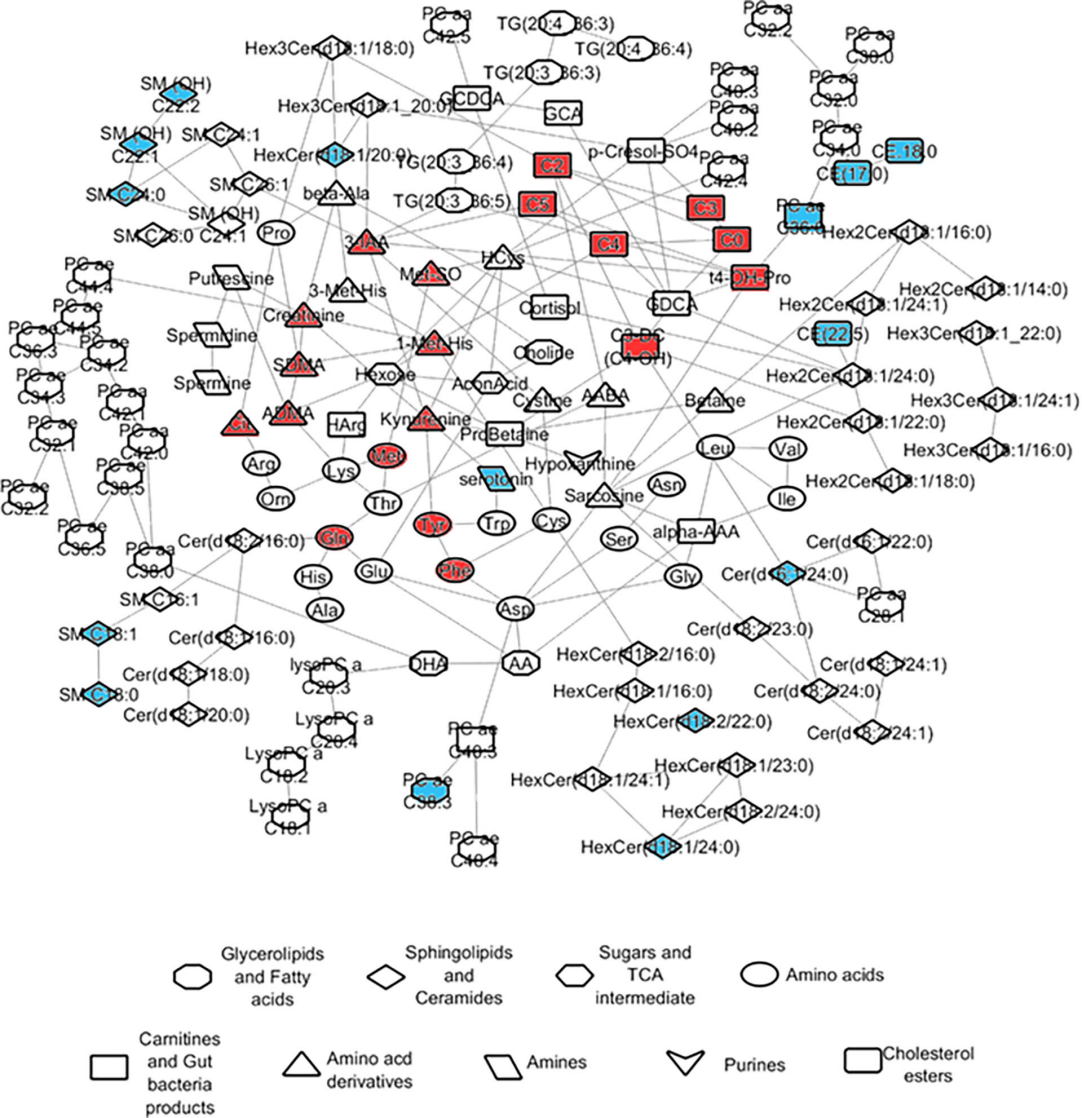
Network representation of metabolite partial correlations based on measurements from the controls. GGM partial correlations that were found significant at FDR <= 0.05 are displayed as edges linking the corresponding metabolite nodes in the network. The colors indicate the abundance levels of the metabolites in ‘alive’ when compared to ‘dead’ after correcting for confounders including post-TB (red/blue corresponding to significantly higher levels in dead or alive respectively).

To characterize the most direct interactions between metabolites based on their levels in the control group, we used Gaussian graphical models (GGMs) partial correlation analysis. The FDR significant partial correlations obtained with the GGMs were displayed as edges linking the respective pairs of metabolites, together giving rise to an overview of the metabolic network in the blood of our recruited control subjects. The analysis projects the differences in measured metabolites in the alive group compared to deceased, with color (**Figure 5**).

## Discussion

**Figure 6:**
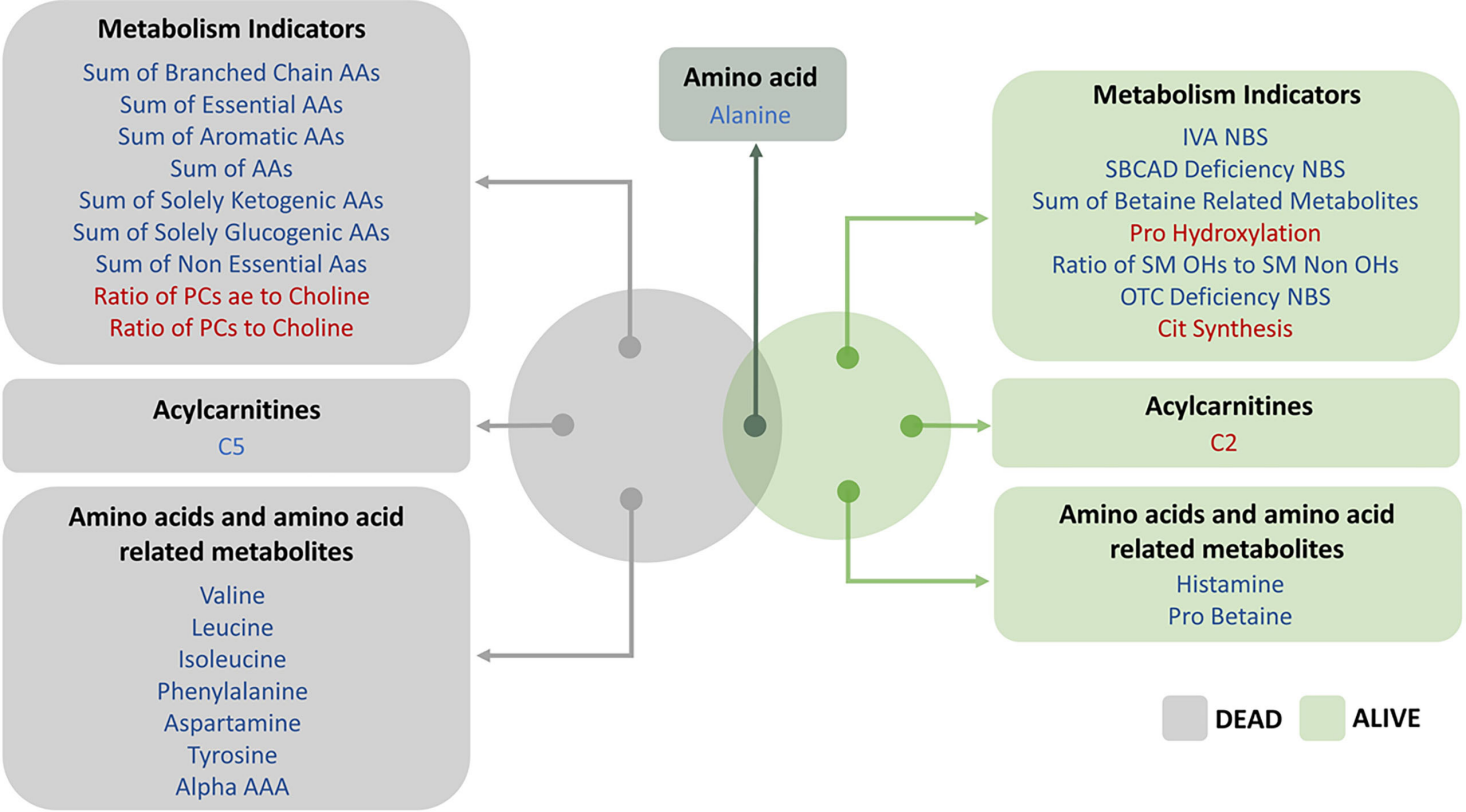
Venn diagram above represents the top FDR significant hits from linear regression model incorporating living status as an ordered categorical variable. The effect (blue/red) represents the offset in the intercept from the controls baseline to post-TB individuals. The blue/red color signifies the metabolites and metabolism indicators that were less/more abundant in the post-TB group respectively.

This study is the first to report an increased mortality in COVID-19 patients who had prior TB infection in a multiethnic cohort. Here we use a large-scale metabolic analysis to further our understanding of the role of metabolic reprogramming post-TB and COVID-19 in the context of severity and mortality and to identify potential biomarkers that can predict the disease progression based on metabolic profile. In this regard, we identify different metabolites that clearly distinguished survived living cases from dead deceased ones in post-TB individuals infected with SARS-CoV-2 (**Figure 6**). These metabolites are included in the metabolism of aspartate, glycine, serine and threonine, leucine, isoleucine and valine (branched-chain amino acids [BCAAs]), lysine, betaine, as well as secondary bile acid metabolism. Interestingly, our analysis also demonstrated serum alanine as an important metabolic biomarker at the interface of severity and mortality in COVID-19 patients who have been exposed to tuberculosis (**Figure 6**).

In severe COVID-19 patients post-TB, alterations occurred in major amino acid metabolic pathways as well as bile acid metabolism. Of these, betaine and its derivatives stand out to be the most prominent in the effect on the mortality and morbidity status in COVID-19 cases who had TB in the past. Betaine, acts acting as a methyl donor, has osmo-protectant properties, and has been recognized for its anti-inflammatory role (Zhao et al., 2018). Our data indicated that anti-inflammatory metabolites including betaine derivatives were significantly reduced in post-TB individuals who suffered from severe symptoms of COVID-19 but survived the infection. In accordance with this, it has been previously shown that betaine significantly mitigated the production of pro-inflammatory cytokines and increased the release of anti-inflammatory cytokines (Zhao et al., 2018). In addition, betaine promoted the conversion of the microglia from M1 to M2 phenotype by decreasing the expression of inducible nitric oxide synthase (Shi et al., 2019). This is all concordant with the classification analysis suggesting a physiological state that is more pro-inflammatory in nature amongst the post-TB group and rather anti-inflammatory in the control group. Our data suggest that betaine and its derivatives did not seem to associate with patients’ survival but appear to associate with COVID-19 severity in post-TB group. Interestingly, vast majority (~92%) of post-TB individuals showed severe SARS-CoV-2 infection. Given that a third of the world’s population is latently infected with *M. tuberculosis* and over 9 million new cases of TB are reported annually new cases of active TB were reported in 2020 (World Health Organization, 2020), betaine could serve as a potential prognostic biomarker of severity in those patients infected with SARS-CoV-2.

In our efforts to explore metabolic biomarkers associated with mortality, our analysis identified a set of anti-inflammatory metabolites including BCAAs (valine, leucine and isoleucine) that were significantly reduced in cases who failed to survive among patients who were previously exposed to TB. BCAAs play a fundamental role in immune cell function (Kelly and Pearce, 2020), as these amino-acids act as an essential source for the synthesis of new effector and protective molecules as well as immune cells (Negro et al., 2008). Moreover, the restriction of these amino-acids increased the susceptibility to pathogen infections by impairing the immune response (Calder, 2006). Furthermore, it has been previously shown that increasing the levels of BCAAs in glial cultures is associated with a shift toward M2 anti-inflammatory macrophage phenotype (From the American Association of Neurological Surgeons et al., 2018). The fact that the post-TB individuals show opposite patterns of BCAAs levels during COVID-19 infection suggests that those individuals may be conferring additional injury due to the promotion of the pro-inflammatory modulators over the anti-inflammatory ones. This is clearly seen in the mobilization of the M1 phenotype in macrophages, within this group of patients, that which may have consequences on the adaptive immune response. Indeed, BCAAs have been shown to maintain T-regulatory cells, and their depletion negatively impact T-regulatory cell function (Ikeda et al., 2017). Another function that can be disrupted within this group of patients and could be a possible cause of mortality, is the critical role of BCAAs in maintaining lymphocytes' function and responsiveness (Calder, 2006). This correlates well with our and others' clinical data showing a striking decrease in the number of lymphocytes in severe and death cases as compared to asymptomatic and mild ones (Tavakolpour et al., 2020; Mohammad et al., 2022). Altogether, our data highlights the potential role of BCAAs to be used as an early prognostic biomarker of possible mortality in post-TB individuals infected with SARS-CoV-2.

Clinically, the consensus on COVID-19 associated morbidity and mortality implies that it is induced by a pro-inflammatory response such that deaths are associated with cytokine storm, or a high pro-inflammatory response associated with auto-immune diseases. In agreement, our study associated mortality and morbidity with a pro-inflammatory metabolic profile. This profile is hypothesized to be a long-term metabolically driven consequence of TB infection rather than a direct immune dysregulation by active TB. Our study reflects the importance of the pro-inflammatory component of the immune system in fighting COVID-19 and hypothesizes that it can be metabolically driven. This work can be extended to study the long-term effect of TB on immunity and metabolism in lung tissue and how it predisposes the lungs to more severe COVID-19 infections. Further research is needed to better understand these changes along with other carnitines and free fatty acids, as reported in COVID-19 metabolomic literature, that may have an important role in the management of vulnerable patients with past pulmonary TB.

Our study is limited by the number of recruited cases and the results presented here need to be confirmed in a larger cohort. The *in-silico* work lays a foundation for wet lab work to further affirm the role of M1 and M2 macrophages in patients who were diagnosed with TB and are infected with SARS-CoV-2. The role of pharmacological agents used in treatment of TB needs to be further investigated in light of long-term metabolic changes that may predispose recovered patients to severe COVID-19 illness.

## Data Availability Statement

The original contributions presented in the study are included in the article/**Supplementary Material**. Further inquiries can be directed to the corresponding authors.

## Ethics Statement

The studies involving human participants were reviewed and approved by Institutional Review Boards (IRBs) of HMC (MRC-05-136) and Qatar University (QU-IRB 1418-EA/20). The patients/participants provided their written informed consent to participate in this study.

## Author Contributions

ID, FC, NA and HY designed and performed the experiments, did the data analysis, and wrote the manuscript. ME and SSc generated the metabolomics data and helped in the analysis. FC, SR, SSa, MA, MK and EF and DB helped in the sampling and collection of patients information. ME and AA designed and supervised the research and edited the manuscript. All authors contributed to the article and approved the submitted version.

## Funding

Work in AMA’s laboratory is supported by the QU internal grant (QUCG-CMED-21/22-2).

## Conflict of Interest

The authors declare that the research was conducted in the absence of any commercial or financial relationships that could be construed as a potential conflict of interest.

## Publisher’s Note

All claims expressed in this article are solely those of the authors and do not necessarily represent those of their affiliated organizations, or those of the publisher, the editors and the reviewers. Any product that may be evaluated in this article, or claim that may be made by its manufacturer, is not guaranteed or endorsed by the publisher.
